# NDP-MSH rescues LPS-induced neuroinflammation, synaptic deficits, and depressive-like behaviors in mice: involvement of MC1R–cAMP/PKA signaling

**DOI:** 10.3389/fphar.2026.1895800

**Published:** 2026-08-05

**Authors:** Shanglan Qu, Xin Peng, Jieyu Ji, Ershu He, Le Jiang, Ruixue Ma, Hanwei Li, Yanjiao Li, Lijuan Li, Hui-Yin Yow, Sharina Hamzah, Zhiting Gong

**Affiliations:** 1 School of Pharmacy, Faculty of Health and Medical Sciences, Taylor’s University, Subang Jaya, Selangor, Malaysia; 2 School of Medicine, Dali University, Dali, Yunnan, China; 3 School of Medicine, Xinjiang College of Science and Technology, Korla, China; 4 Department of Pharmaceutical Life Sciences, Faculty of Pharmacy, Universiti Malaya, Kuala Lumpur, Malaysia

**Keywords:** depressive-like behaviors, MC1R, NDP-MSH, neuroinflammation, synaptic deficits

## Abstract

**Introduction:**

Inflammatory processes contribute significantly to the pathophysiology of depression. Although the melanocortin system is well known to regulate inflammation, the specific contribution of melanocortin 1 receptor (MC1R) and its endogenous ligand, α-melanocyte-stimulating hormone (α-MSH), to inflammation-associated depression remains unclear.

**Methods:**

Systemic lipopolysaccharide (LPS) administration was used to establish an inflammation-associated depression model in mice. Depressive-like behaviors, synaptic functions, and metabolic alterations were evaluated using behavioral tests, patch-clamp recordings, and untargeted metabolomic profiling. To examine the functional involvement of MC1R, adeno-associated virus (AAV)-mediated selective *Mc1r* overexpression was performed in the medial prefrontal cortex (mPFC).

**Results:**

LPS administration induced depressive-like behaviors in mice, accompanied by microglial activation and a significant reduction in MC1R expression in the prefrontal cortex (PFC). Treatment with MC1R endogenous ligand α-MSH mimetic Nle4-DPhe7-α-MSH (NDP-MSH) markedly attenuated LPS-induced depressive-like behaviors, enhanced MC1R, postsynaptic density protein 95 (PSD95), glutamate receptor 1 (GluA1) and protein kinase A (PKA) phosphorylation. PKA inhibitor H89-mediated inhibition of the cyclic adenosine monophosphate/protein kinase A (cAMP/PKA) pathway partially but robustly abolishes the behavioral, anti-inflammatory, and synaptic protective effects of NDP-MSH. Additionally, untargeted metabolomics confirmed that NDP-MSH effectively corrected LPS-induced metabolic dysregulation, a therapeutic effect that was robustly suppressed by H89; this metabolic remodeling was closely associated with purine metabolism, pantothenate, and coenzyme A biosynthesis. Critically, AAV-mediated *Mc1r* overexpression in the mPFC was sufficient to rescue LPS-induced depressive-like phenotypes.

**Conclusion:**

This study highlights MC1R-related signaling in the PFC as an important contributor to inflammation-associated depression and suggests that NDP-MSH alleviates inflammation-associated depressive-like behaviors in association with melanocortin signaling involving MC1R and downstream cAMP/PKA activation.

## Graphical Abstract

**Figure:**
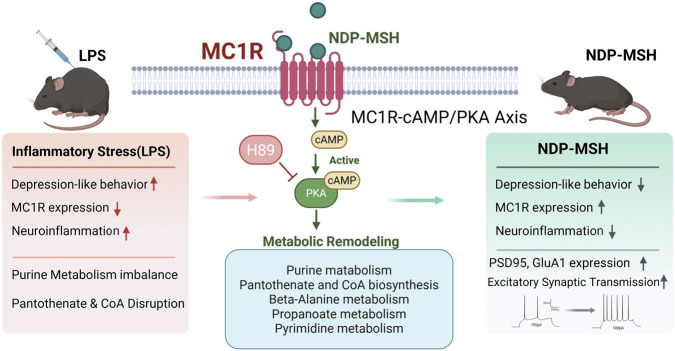

## Introduction

Depression affected more than 332 million people worldwide in 2021 (GBD 2021 Diseases and Injuries Collaborators, 2024) and is characterized by persistently low mood, diminished interest or pleasure in most activities, and a broad spectrum of cognitive, emotional, and somatic symptoms (Herrman et al., 2022). Its pathogenesis is highly complex, with neuroinflammation recognized as a key pathological contributor. Pro-inflammatory cytokines and microglial activation play critical roles in the development of depressive disorders (Yirmiya, 2024). Clinical evidence indicates that C-reactive protein (CRP), interleukin (IL)-12, and soluble IL-2 receptor (sIL-2R) are not only elevated in mean levels but also show markedly reduced variability in patients with depression, suggesting a more uniform inflammatory profile in patients with depression (Osimo et al., 2020). Current pharmacotherapy primarily targets dysregulation within noradrenergic, serotonergic, dopaminergic, or glutamatergic neurotransmission. Despite these therapeutic strategies, approximately 30% of patients fail to respond to standard first-line treatments (Kim et al., 2024).

The melanocortin system plays an essential role in modulating inflammatory responses, largely through its receptor-mediated anti-inflammatory and neuroprotective mechanisms (Getting, 2002; Catania et al., 2010). Melanocortin 1 receptor (MC1R), a G protein-coupled receptor belonging to the melanocortin receptor family, is highly expressed in the skin, where it regulates melanogenesis and pigmentation through interactions with endogenous ligands. Subsequent studies revealed that MC1R also modulates inflammatory responses through its broad expression in immune cells, including monocytes and macrophages (Suominen et al., 2025). Recently, therapeutic agents targeting MC1R, including afamelanotide, dersimelagon, and bremelanotide, have expanded their applications beyond pigmentation disorders, demonstrating potential therapeutic efficacy in systemic sclerosis, neuroinflammation, rheumatoid arthritis, and fibrosis-related diseases (Mun et al., 2023).

MC1R activity is closely associated with the binding of its endogenous ligand α-melanocyte-stimulating hormone (α-MSH), which exerts antipyretic, antimicrobial, anti-inflammatory, and immunomodulatory effects. Notably, afamelanotide, a well-known α-MSH analog, is the first approved drug for erythropoietic protoporphyria, where it enhances melanogenesis to provide photoprotection (Luger and Böhm, 2015). In addition, α-MSH suppresses the expression of pro-inflammatory cytokines and adhesion molecules, promotes anti-inflammatory cytokine production, and reduces the release of other inflammatory mediators such as nitric oxide and prostaglandins, thereby regulating immune cell activity (Wang et al., 2019; Dinparastisaleh and Mirsaeidi, 2021).

However, α-MSH is chemically unstable, highly susceptible to proteolytic degradation, and becomes undetectable in plasma within 30–90 min after intravenous administration (Rudman et al., 1983). A modified analog, Nle4-D-Phe7-α-MSH (NDP-MSH), exhibits improved stability and stronger affinity for MC1R (Yang et al., 1997). NDP-MSH has demonstrated neuroprotective effects across various neuroinflammatory models (Mykicki et al., 2016; Wu et al., 2019; Fu et al., 2020; Lu et al., 2024). However, the role of NDP-MSH in depression remains unclear.

In this study, the role of MC1R and its agonist, NDP-MSH was investigated in lipopolysaccharide (LPS)-induced inflammation-associated depression. Our data demonstrate that NDP-MSH significantly attenuated LPS-induced depressive-like phenotypes, reduced microglial activation and interleukin-1β (IL-1β) levels in the prefrontal cortex, and restored synaptic deficits-effects that were partially reversed by protein kinase A (PKA) inhibition. Furthermore, *Mc1r* overexpression in the medial prefrontal cortex (mPFC) markedly ameliorates LPS-induced depressive-like behaviors in mice. These findings elucidate a mechanistic link between MC1R-related melanocortin signaling and the mitigation of LPS-induced neuroinflammation and depressive-like behaviors via cyclic adenosine monophosphate/PKA (cAMP/PKA) signaling, highlighting the therapeutic potential of NDP-MSH-based intervention for inflammation-associated depression.

## Materials and methods

### Animals

Male C57BL/6J mice were purchased from SPF Biotechnology Co., Ltd. (Beijing, China) and housed at the Experimental Animal Center of Dali University. Animals were maintained under controlled conditions (22 °C ± 1 °C) with a 12-h light/dark cycle (lights on from 8:00 to 20:00), with *ad libitum* access to food and water. Mice were group-housed (4-6 per cage). Behavioral experiments were initiated when mice were 8–10 weeks of age. For each experiment, mice of the same age and sex were purchased in a single batch to minimize variability.

All animal procedures were conducted in accordance with the ARRIVE guidelines and were approved by the Animal Care and Use Committee of Dali University (Approval No. MECDU-202203-05).

### Depression-like behavior induction and drug administrations

Mice were treated by intraperitoneal injection once daily for seven consecutive days except in the AAV experiment. For mice assigned to treatment regimens containing more than one agent, all drugs were mixed together in saline immediately before administration and delivered simultaneously as a single intraperitoneal injection. Saline was used as the vehicle for all treatment groups, and vehicle-treated mice received an equivalent volume of saline. In the AAV experiment, both the mCherry control group and the Mc1r overexpression group received 6 consecutive days of LPS treatment.

Behavioral tests were performed during the ongoing treatment period. The sucrose preference test (SPT) was conducted on days 5 and 6, and the open field test (OFT) was performed on day 7, 2 h after the daily injection (In the AAV experiment, OFT was performed on day 6, 2 h after the daily injection). Treatment regimens were as follows: vehicle-treated mice received equivalent volumes of saline; the LPS group received lipopolysaccharide (LPS; 1 mg/kg; L2880, Sigma-Aldrich, United States); the LPS + NDP-MSH group received LPS (1 mg/kg) together with NDP-MSH (2 mg/kg; HY-N2466, MedChemExpress, United States); the LPS + NDP-MSH + H89 group received LPS (1 mg/kg), NDP-MSH (2 mg/kg), and the PKA inhibitor H89 (10 mg/kg; HY-15979A, MedChemExpress, United States); and the H89-alone group received H89 (10 mg/kg).

LPS (L2880, Sigma-Aldrich, United States) was dissolved in double-distilled water to prepare a stock solution (5 μg/μL) and stored at 4 °C. Prior to use, LPS was diluted in saline and administered i.p. at a dose of 1 mg/kg.

NDP-MSH (HY-N2466, MedChemExpress, United States) was dissolved in double-distilled water to prepare a stock solution (5 μg/μL), aliquoted, and stored at −20 °C. Before administration, it was diluted in saline and injected i. p. at 2 mg/kg (Dorr et al., 1988).

H89 (HY-15979A, MedChemExpress, United States) was dissolved in double-distilled water with ultrasonic heating at 80 °C to obtain a stock solution (5 μg/μL), aliquoted, and stored at −20 °C. It was diluted in saline before i. p. injection at 10 mg/kg.

### Behavioral tests

Mice were subjected to the sucrose preference test (SPT), followed by the open field test (OFT). For rAAV-injected mice, the forced swim test (FST) was additionally performed at the end of the behavioral cohort. To minimize potential bias, drug administration and behavioral testing/scoring were performed by different investigators. Behavioral videos were coded before analysis, and the investigator responsible for behavioral scoring was blinded to treatment allocation and had no access to drug administration information until all behavioral analyses were completed.

### Sucrose preference test

The sucrose preference test (SPT) was conducted using a two-bottle free-choice paradigm as previously described (Ali et al., 2020). Mice were deprived of food and water for 24 h. The following day, mice were allowed free access to two bottles for 24 h, one containing tap water and the other containing 1% sucrose solution. Bottle positions were switched after 12 h to avoid side preference. Fluid consumption was measured, and sucrose preference was calculated as:

$$\text{Sucrose preference }\left(\%\right)=\text{sucrose intake}/\left(\text{water}+\text{sucrose intake}\right)\times 100$$

### Open field test

The open field test (OFT) was performed as previously described (He et al., 2024). The apparatus consisted of a 45 × 45 × 30 cm arena under 150 lux illumination. Mice were allowed to acclimate to the testing room for at least 1 h before testing. Each mouse was placed in the center of the arena and recorded for 15 min. Behavioral data were collected using Visual Track software (Shanghai XinRuan Information Technology Co. Ltd, Shanghai, China). The arena was cleaned with 75% ethanol between animals. Room temperature was maintained at 25 °C.

### Forced swim test

The forced swim test (FST) was performed with minor modifications based on Zheng et al. (2022). The forced swim test (FST) was conducted 24 h after the final LPS injection. Mice were placed individually into transparent acrylic cylinders (15 cm diameter, 30 cm height) filled with water (23 °C–25 °C) to a depth sufficient to prevent tail or hindlimb contact with the bottom. Following 1 h acclimation period, mice were subjected to 6-min test session. Immobility time during the final 5 min was recorded using Visual Track software. Water was replaced after each session before the next mouse.

### Immunofluorescence

After completion of behavioral tests, mice were deeply anesthetized with isoflurane and perfused with PBS followed by 4% paraformaldehyde (PFA). Brains were post-fixed overnight in 4% PFA and cryoprotected in 20% and 30% sucrose solutions. Coronal sections (30 μm) containing the PFC were collected and stored at 4 °C. Sections were blocked in PBS containing 0.5% Triton X-100, 10% goat serum, and 0.2% skim milk for 1 h at room temperature and incubated overnight with primary antibody (rabbit anti-Iba1, 1:500; Wako, Cat#019-19741). After washing, sections were incubated with Alexa Fluor 488-conjugated secondary antibody (1:400; CST, Cat#4412S) for 1 h in the dark, followed by Hoechst 33342 nuclear staining. Images were acquired using a Zeiss fluorescence microscope (Zen 2.3) with ×10 objective. Quantification of Iba1-positive cell number, fluorescence intensity, and soma diameter was performed using ImageJ in a blinded manner.

### Western blot

PFC tissues were homogenized in ice-cold RIPA buffer (Boster, Wuhan, China; cat. no. AR0102-100) supplemented with protease inhibitor (Roche, Basel, Switzerland; cat. no. 11836153001) and phosphatase inhibitor (MedChemExpress, Monmouth Junction, NJ, United States; cat. no. HY-K0021, cat. no. HY-K0023). After incubation on ice for 30 min, lysates were centrifuged at 12,000 × g for 10 min at 4 °C. Protein concentration was determined using a bicinchoninic acid (BCA) assay (Beyotime, Shanghai, China; cat. no. P0010S). Equal amounts of protein (30 μg) were separated by SDS-PAGE and transferred to PVDF membranes. Membranes were blocked with 5% non-fat milk and then incubated with primary antibodies overnight at 4 °C, followed by HRP-conjugated secondary antibodies for 1 h at room temperature. Protein bands were visualized using a ChemiDoc XRS system (Bio-Rad Laboratories, Inc., United States) and quantified with Image Lab software. Target protein levels were normalized to GAPDH.

Primary and secondary antibodies were prepared as follow:

Primary antibodies and dilutions: MC1R (ThermoFisher, #PA5-75342, Rabbit, 1:1000); Phospho- (Ser/Thr) PKA Substrate Antibody (pPKA) (Cell Signaling Technology, #9621S, Rabbit, 1:1000); GluA1 (Cell Signaling Technology, #13185T, Rabbit, 1:1000); PSD95 (Cell Signaling Technology, #3450T, Rabbit, 1:1000); GAPDH (Cell Signaling Technology, #2118S, Rabbit, 1:1000).

Secondary antibodies: Anti-Rabbit IgG, HRP-linked (Cell Signaling Technology, #7074S, 1:5000).

### Golgi staining

Golgi staining was performed using the FD Rapid GolgiStain Kit (FD NeuroTechnologies, United States; cat. no. PK401) according to the manufacturer’s instructions. Brains were impregnated in Solutions A/B for 2 weeks, transferred to Solution C for 72 h, sectioned at 150 μm, stained, dehydrated, cleared, and imaged using a Zeiss fluorescence microscope (Zen 2.3) with ×40 objective.

### Enzyme-linked immunosorbent assay

IL-1β levels were measured using a high-sensitivity mouse IL-1β ELISA kit (Elabscience, Wuhan, China; cat. no. E-EL-M0037). Tissue homogenates were prepared in PBS (1:9, w/v). Assays were performed according to the manufacturer’s instructions, and absorbance was measured using a microplate reader.

### Brain stereotaxic injection

To achieve long-term, cell-type-specific Mc1r overexpression in the mouse brain, adeno-associated virus vectors were utilized. AAV2/9-CaMKIIa-mCherry-P2A-*Mc1r*-3×Flag-WPRE (titer: 2.70 × 10^^12^ vg/mL) and its control vector AAV2/9-CaMKIIa-mCherry-P2A-3×Flag-WPRE (diluted 10-fold to a final working titer of 2.06 × 10^^12^ vg/mL) were packaged by OBiO Technology Corp., Ltd. (Shanghai, China) and stored at −80 °C. Mice were anesthetized with isoflurane (induction at 3%–4%, maintained at 1.5%–2% in air) and secured in a stereotaxic frame. AAV vectors were then bilaterally microinjected into the mPFC. The viral suspension (0.5 μL/site) was infused via a Hamilton syringe at a rate of 0.18 μL/min based on the following coordinates relative to bregma: AP +1.8 mm, ML ±0.24 mm, and DV −1.75 mm. Following infusion, the needle was maintained in place for an additional 3 min to allow adequate diffusion before gradual withdrawal. After recovery from anesthesia, mice were returned to their home cages with *ad libitum* access to food and water. Behavioral and biochemical experiments were performed 21 days post-injection to ensure robust transgene expression.

### Whole-cell patch-clamp recordings

Acute coronal brain slices (300 μm thick) containing the medial prefrontal cortex (mPFC) were prepared from 6–8-week-old male mice. Live brain slices were acutely prepared from mouse brains as previously described (Gao et al., 2019). Briefly, mice were decapitated, and the brains were rapidly isolated into ice-cold high-sucrose cutting solution bubbled with 95% O_2_ and 5% CO_2_. Slices were cut using a vibrating microtome. Following sectioning, slices were transferred to oxygenated artificial cerebrospinal fluid (ACSF) at 35 °C for a recovery period, and subsequently maintained at room temperature (22 °C–24 °C) prior to pharmacological treatment and recording.

To evaluate the pharmacological effects of NDP-MSH, slices were pre-incubated in oxygenated ACSF containing 1 μM NDP-MSH for at least 30 min before the commencement of the first recording. For electrophysiological recordings, a single treated slice was transferred to a recording chamber and continuously perfused with the drug-containing ACSF at room temperature (22 °C–24 °C). Whole-cell current-clamp recordings were performed on mPFC neurons, situated predominantly within Layers V/VI. Upon establishing the whole-cell configuration (break-in), a 10-min equilibration period was strictly maintained for each patched neuron to ensure stable configuration and signal baseline before data acquisition.

Action potentials and intrinsic excitability parameters were evoked by a series of step current injections. Rheobase and spike numbers were quantified using Clampfit 10.7 software (Molecular Devices, United States) with a MultiClamp 700B amplifier. The chemical compositions of the solutions were as follows:

High-sucrose artificial cerebrospinal fluid (cutting solution): 2.5 mM KCl, 1.2 mM NaH_2_PO_4_, 26 mM NaHCO_3_, 252 mM sucrose, 6 mM MgSO_4_, 0.5 mM CaCl_2_, and 10 mM glucose; osmolarity 300–320 mOsm/L; pH 7.3–7.4.

ACSF: 124 mM NaCl, 2.5 mM KCl, 2 mM CaCl_2_, 2 mM MgSO_4_, 25 mM NaHCO_3_, 1 mM NaH_2_PO_4_, and 10 mM glucose; osmolarity 280–300 mOsm/L; pH 7.3–7.4.

Potassium gluconate–based internal solution (for patch electrodes): 130 mM potassium gluconate, 5 mM NaCl, 15 mM KCl, 0.4 mM EGTA, 10 mM HEPES, 4 mM Mg-ATP, and 0.3 mM Na_2_-GTP; osmolarity 280–320 mOsm/L; pH 7.3–7.4.

### Untargeted metabolomics analysis

LC–MS/MS Data Acquisition: Untargeted metabolomic profiling was performed using an ultra-high-performance liquid chromatography–tandem mass spectrometry (UHPLC–MS/MS) system consisting of a Q Exactive™ HF-X Orbitrap mass spectrometer coupled to a UHPLC platform (Thermo Fisher Scientific, United States). Data acquisition was conducted with technical support from Majorbio Bio-Pharm Technology Co., Ltd. (Shanghai, China). Chromatographic separation was achieved using an HSS T3 column (100 × 2.1 mm, 1.8 μm). The injection volume was 3 μL, with a flow rate of 0.40 mL/min and a column temperature of 40 °C. Mobile phase A consisted of water/acetonitrile (95:5, v/v) containing 0.1% formic acid, and mobile phase B consisted of acetonitrile/isopropanol/water (47.5:47.5:5, v/v/v) containing 0.1% formic acid. Mass spectrometric data were acquired in both positive and negative electrospray ionization modes over an m/z range of 70–1050. The sheath gas flow rate was set to 50 psi, auxiliary gas flow rate to 13 psi, and auxiliary gas heater temperature to 425 °C. The spray voltage was set to +3.5 kV in positive mode and −3.5 kV in negative mode, and the ion transfer tube temperature was maintained at 325 °C. Data were collected using data-dependent acquisition (DDA) mode with a full MS resolution of 60,000 and MS/MS resolution of 7,500. Normalized collision energy (NCE) was set at 20, 40, and 60 eV.

Data Processing and Quality Control: Raw LC–MS/MS data were processed using standard workflows for baseline filtering, peak identification, peak integral, retention time correction, and peak alignment. Quality control (QC) samples were used to assess analytical stability and reproducibility throughout the run and were excluded from downstream differential metabolite analysis. To reduce the errors caused by sample preparation and instrument instability, the response intensities of the sample mass spectrometry peaks were normalized using the sum normalization method, to obtain the normalized data matrix. Meanwhile, the variables of QC samples with relative standard deviation (RSD) > 30% were excluded and log10 logarithmicized, to obtain the final data matrix for subsequent analysis. Initial metabolomic data processing, preliminary candidate metabolite screening, and partial visualization were performed using the Majorbio Cloud platform. Downstream correlation analyses were performed independently in R.

Differential Metabolite Analysis: Differential metabolite analysis was conducted in R using the limma package. Samples were grouped as Control (Saline), LP (LPS-treated), NDP (LPS + NDP-MSH), and LNH (LPS + NDP-MSH + H89), with additional reference groups included where appropriate. For each metabolite, a linear model was fitted using a group-wise design matrix without an intercept. The following contrasts were specified: LPS vs. CTRL; NDP vs. LPS; LNH vs. NDP; NDP vs. CTRL; LNH vs. CTRL; LNH vs. LPS. Empirical Bayes moderation was applied to stabilize variance estimates across metabolites. Resulting p values were adjusted for multiple testing using the Benjamini–Hochberg false discovery rate (FDR) procedure. Metabolites with FDR <0.20 and |log_2_ fold change| ≥ 0.15 were considered significantly altered and were used for downstream visualization and summary analyses.

Reversal and Inhibition Pattern Analysis: To assess whether drug treatment reversed disease-associated metabolic alterations, log_2_ fold changes derived from the LPS vs. Control contrast were compared with those from the NDP vs. LPS contrast across all detected metabolites. Spearman correlation analysis was performed using complete observations only. Similarly, to evaluate whether inhibitor treatment attenuated drug-induced metabolic changes, log_2_ fold changes from the NDP vs. LPS contrast were compared with those from the LNH vs. NDP contrast.

Pathway Enrichment Analysis: Significantly altered metabolites were subjected to pathway enrichment and topology analysis based on KEGG annotations using MetaboAnalyst (v5.0). Pathway significance was assessed using enrichment *p* values, and pathway impact scores were calculated according to network topology measures. Results were visualized as bubble plots, with pathway impact on the x-axis and −log_10_(p value) on the y-axis.

### Statistical analysis

Data were analyzed using GraphPad Prism version 10.1 from at least three independent experiments. Results were presented as mean ± SEM. Normality was assessed using the Shapiro-Wilk test. For normally distributed data, two-group comparisons were carried out using Student’s unpaired two-tailed t-test when variances were equal. Multiple-group comparisons were performed by one-way ANOVA followed by Tukey’s *post hoc* test and Uncorrected Fisher’s LSD *post hoc* test, as variances were homogeneous. A *p*-value <0.05 was considered statistically significant (*p < 0.05, **p < 0.01, ***p < 0.001, ****p < 0.0001).

## Results

### MC1R expression is reduced in the PFC of LPS-induced inflammation-associated depressive-like behaviors

**FIGURE 1:**
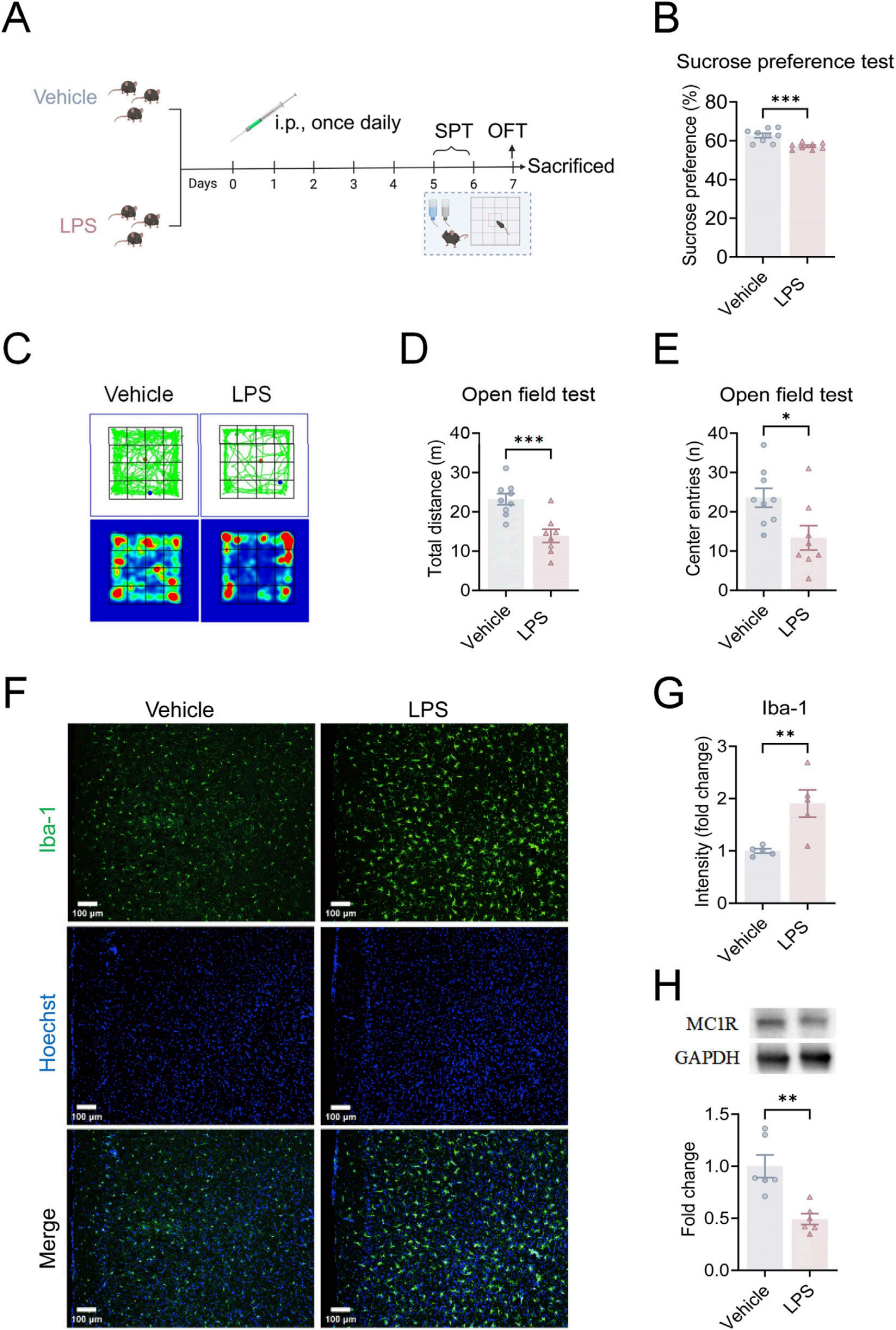
LPS induces depressive-like behaviors and alters microglial activity and MC1R expression in mice. **(A)** Schematic timeline of the experimental procedure. **(B)** Sucrose preference in the sucrose preference test (SPT), LPS-treated mice exhibited a significantly reduced sucrose preference compared with vehicle-treated controls (two-tailed unpaired Student’s t-test; t_(15)_ = 4.110, *p <* 0.001) (Vehicle, n = 9; LPS, n = 8). **(C)** Representative locomotor traces in the open-field test (OFT). **(D)** Total distance traveled in the OFT, LPS-treated mice significantly decreased locomotor activity relative to vehicle-treated controls (two-tailed unpaired Student’s t-test; t_(15)_ = 4.255, *p <* 0.001) (Vehicle, n = 9; LPS, n = 8). **(E)** Number of center entries in the OFT, LPS-treated mice entered the center zone significantly fewer times than vehicle-treated controls (two-tailed unpaired Student’s t-test; t_(15)_ = 2.624, *p <* 0.05) (Vehicle, n = 9; LPS, n = 8). **(F)** Representative immunofluorescence images of Iba-1 staining in the mPFC, Scale bar = 100 µm. **(G)** Quantification of relative Iba-1 fluorescence intensity, increased intensity of Iba-1 in LPS-treated mice (two-tailed unpaired Student’s t-test; t_(8)_ = 3.440, *p < 0.01*) (n = 5 mice per group). **(H)** Western blot analysis of MC1R protein expression in the PFC between LPS-treated mice and vehicle-treated controls (two-tailed unpaired Student’s t-test; t_(10)_ = 4.217, *p <* 0.01) (n = 6 mice per group). All the values were normalized with loading control GAPDH. Data are presented as mean ± SEM. Statistical significance: *p < 0.05, **p < 0.01, ***p < 0.001. Source: BioRender.com.

The LPS-induced mouse model of depressive-like behavior is widely used to study inflammation-associated depression. To establish the relationship between depression and MC1R, mice received intraperitoneal (i.p.) LPS injections for 7 consecutive days following the schedule illustrated in Figure 1A. Compared with vehicle-treated controls, LPS-treated mice exhibited a significant reduction in sucrose preference in the SPT (Figure 1B). In the OFT, representative locomotor traces and heatmaps are shown in Figure 1C. LPS administration led to a decrease in total distance traveled (Figure 1D) and fewer center entries (Figure 1E). Furthermore, immunofluorescence analysis revealed enhanced Iba-1 staining intensity in the PFC of LPS-treated mice, indicating robust microglial activation (Figures 1F,G). These results collectively demonstrate that LPS successfully induced depressive-like behaviors and neuroinflammation in mice.

Moreover, Western blot analysis demonstrated that MC1R protein levels in the PFC were significantly downregulated following LPS administration (Figure 1H). Together, these findings demonstrate that repeated peripheral LPS exposure induces inflammation-associated depressive-like behaviors, particularly reduced sucrose preference, together with decreased locomotor and exploratory activity. These changes were accompanied by microglial activation and a reduction in MC1R expression in the PFC. These observations implicate PFC MC1R downregulation as a potential contributor to LPS-induced inflammation-associated depressive-like phenotypes.

### Activation of MC1R by NDP-MSH rescues LPS-induced depressive-like behaviors and synaptic deficits

**FIGURE 2:**
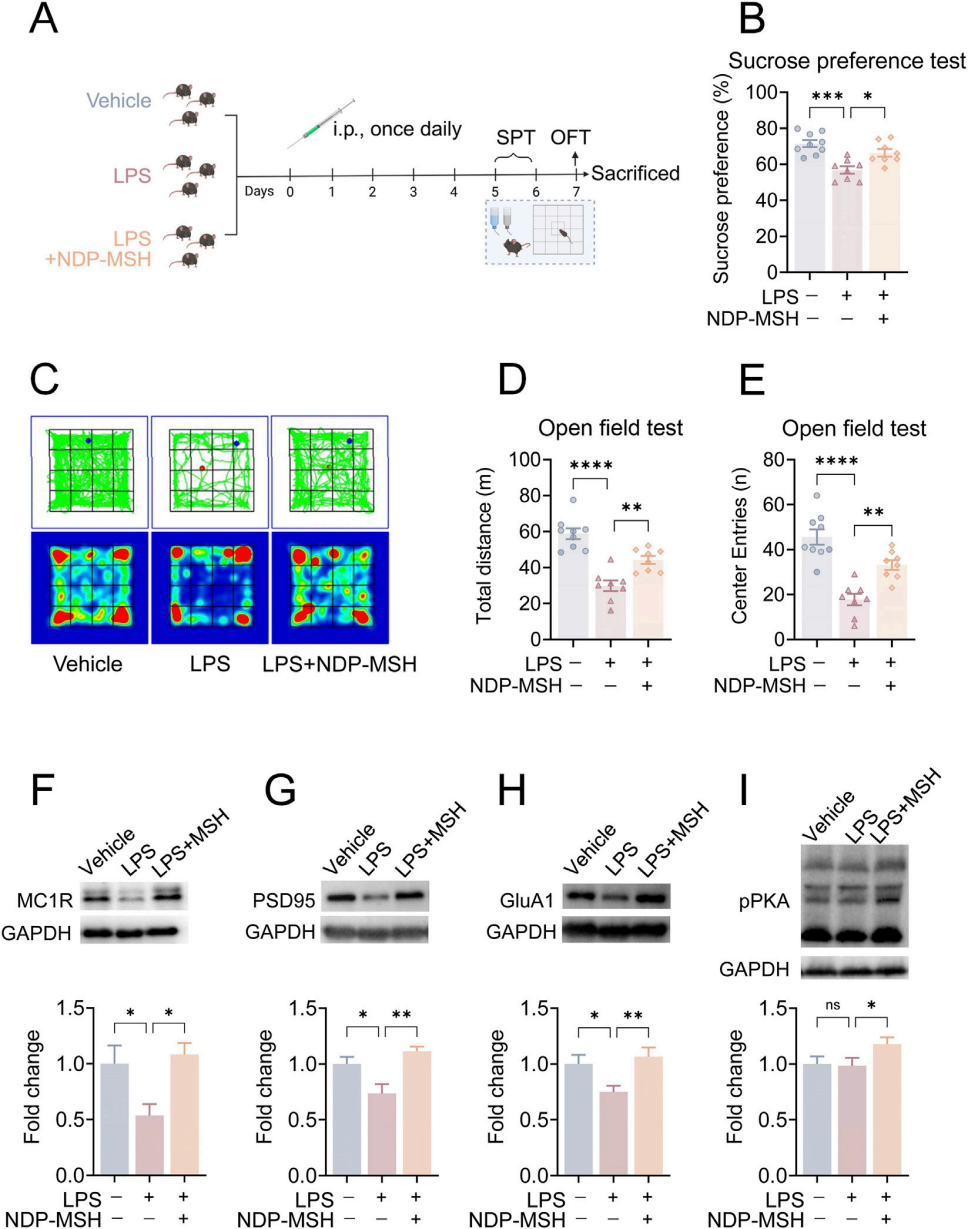
NDP-MSH ameliorates LPS-induced inflammation-associated depressive-like behaviors and synaptic deficits, restores MC1R expression in the PFC. **(A)** Experimental timeline illustrating the LPS and NDP-MSH administration schedule and behavioral testing paradigm. **(B)** Sucrose preference in the SPT. NDP-MSH significantly reversed the LPS-induced reduction in sucrose preference (One-way ANOVA, F_(2, 22)_ = 13.33, *p* < 0.001, followed by Tukey’s multiple comparisons test. Vehicle vs. LPS, q_(22)_ = 7.235, *p* < 0.001; LPS vs. LPS + NDP-MSH, q_(22)_ = 4.576, *p* < 0.05; Vehicle, n = 9; LPS, n = 8; LPS + NDP-MSH, n = 8). **(C)** Representative locomotor traces in the OFT. **(D)** Total distance traveled in the OFT. NDP-MSH attenuated the LPS-induced reduction in total distance traveled (One-way ANOVA, F_(2, 22)_ = 27.68, *p* < 0.0001, followed by Tukey’s multiple comparisons test. Vehicle vs. LPS, q_(22)_ = 10.51, *p* < 0.0001; LPS vs. LPS + NDP-MSH, q_(22)_ = 5.066, *p* < 0.01; Vehicle, n = 9; LPS, n = 8; LPS + NDP-MSH, n = 8). **(E)** Number of center entries in the OFT. NDP-MSH significantly attenuated the LPS-induced reduction in center entries (One-way ANOVA, F_(2, 22)_ = 24.18, *p* < 0.0001, followed by Tukey’s multiple comparisons test. Vehicle vs. LPS, q_(22)_ = 9.831, *p* < 0.0001; LPS vs. LPS + NDP-MSH, q_(22)_ = 5.283, *p* < 0.01; Vehicle, n = 9; LPS, n = 8; LPS + NDP-MSH, n = 8). **(F)** Western blot analysis of MC1R protein expression in the PFC. NDP-MSH significantly increased MC1R protein levels in LPS-treated mice (One-way ANOVA, F_(2, 6)_ = 5.477, *p* < 0.05, followed by Fisher’s LSD test for planned pairwise comparisons. Vehicle vs. LPS, t_(6)_ = 2.605, *p* < 0.05; LPS vs. LPS + NDP-MSH, t_(6)_ = 3.071, *p* < 0.05; n = 3 per group). All the values were normalized with loading control GAPDH. **(G)** Western blot and quantification showing significantly increased PSD95 protein levels in the NDP-MSH group compared with the LPS group (One-way ANOVA, F_(2, 15)_ = 8.682, *p* < 0.01, followed by Fisher’s LSD test for planned pairwise comparisons. Vehicle vs. LPS, t_(15)_ = 2.842, *p* < 0.05; LPS vs. LPS + NDP-MSH, t_(15)_ = 4.060, *p* < 0.01; n = 6 per group). All the values were normalized with loading control GAPDH. **(H)** Western blot and quantification showing significantly increased GluA1 protein levels in the NDP-MSH group compared with the LPS group (One-way ANOVA, F_(2, 15)_ = 5.127, *p* < 0.05, followed by Fisher’s LSD test for planned pairwise comparisons. Vehicle vs. LPS, t_(15)_ = 2.401, *p* < 0.05; LPS vs. LPS + NDP-MSH, t_(15)_ = 3.035, *p* < 0.01; n = 6 per group). All the values were normalized with loading control GAPDH. **(I)** Western blot and quantification showing significantly increased pPKA protein levels in the NDP-MSH group compared with the LPS group (One-way ANOVA, F_(2,24)_ = 2.628, *p* = 0.0929, followed by Fisher’s LSD test for planned pairwise comparisons. Vehicle vs. LPS, t_(24)_ = 0.1813, *p* = 0.8577; LPS vs. LPS + NDP-MSH, t_(24)_ = 2.070, *p* < 0.05) (n = 9 per group). All the values were normalized with loading control GAPDH. Data are presented as mean ± SEM. Statistical significance: *p < 0.05, **p < 0.01, ***p < 0.001, ****p < 0.0001. Source: BioRender.com.

α-MSH is the endogenous ligand of MC1R, but its use is limited by its short half-life and instability (Dall’Olmo et al., 2023; Ng and Taylor, 2023; Rinne et al., 2023). NDP-MSH, a more stable α-MSH analogue, can effectively activate MC1R with enhanced metabolic stability (Rennalls et al., 2010; Todorovic et al., 2016). To further investigate the role of MC1R in LPS-induced depressive-like behaviors, mice were treated with NDP-MSH following the schedule shown in Figure 2A.

Behavioral assessments indicated that the administration of NDP-MSH significantly reversed LPS-induced behavioral despair and locomotor impairments. Specifically, NDP-MSH treatment markedly restored the sucrose preference in the SPT, which had been reduced by LPS (Figure 2B). In the OFT, representative exploratory tracks and heatmaps illustrated a clear recovery of locomoting activity in the combination group (Figure 2C). Quantitative analysis confirmed that NDP-MSH significantly increased both the total distance traveled (Figure 2D) and the number of center entries (Figure 2E) compared with the LPS-only group. These findings demonstrate that MC1R activation effectively rescues LPS-induced depressive-like behaviors in mice.

To further elucidate the underlying molecular mechanisms, we examined the expression of MC1R, synaptic plasticity-associated proteins, and downstream signaling molecules in the PFC using Western blot analysis. As expected, NDP-MSH treatment successfully prevented the LPS-induced downregulation of MC1R protein levels (Figure 2F). Concurrently, the expression levels of key postsynaptic proteins, including PSD95 (Figure 2G) and GluA1 (Figure 2H), were significantly reduced in the LPS group but effectively restored to near-baseline levels upon NDP-MSH administration. Furthermore, we assessed the phosphorylation of protein kinase A (pPKA), a classical downstream effector of MC1R activation. While LPS exposure alone did not significantly alter pPKA levels compared to the vehicle controls, the addition of NDP-MSH led to upregulation of pPKA expression in the PFC (Figure 2I).

Collectively, these results suggest that MC1R activation by NDP-MSH reverses LPS-induced inflammation-associated depressive-like behaviors, potentially through the upregulation of the pPKA signaling pathway and the preservation of synaptic protein expression.

### PKA inhibition by H89 abolishes the neuroprotective and antidepressant effects of NDP-MSH

**FIGURE 3:**
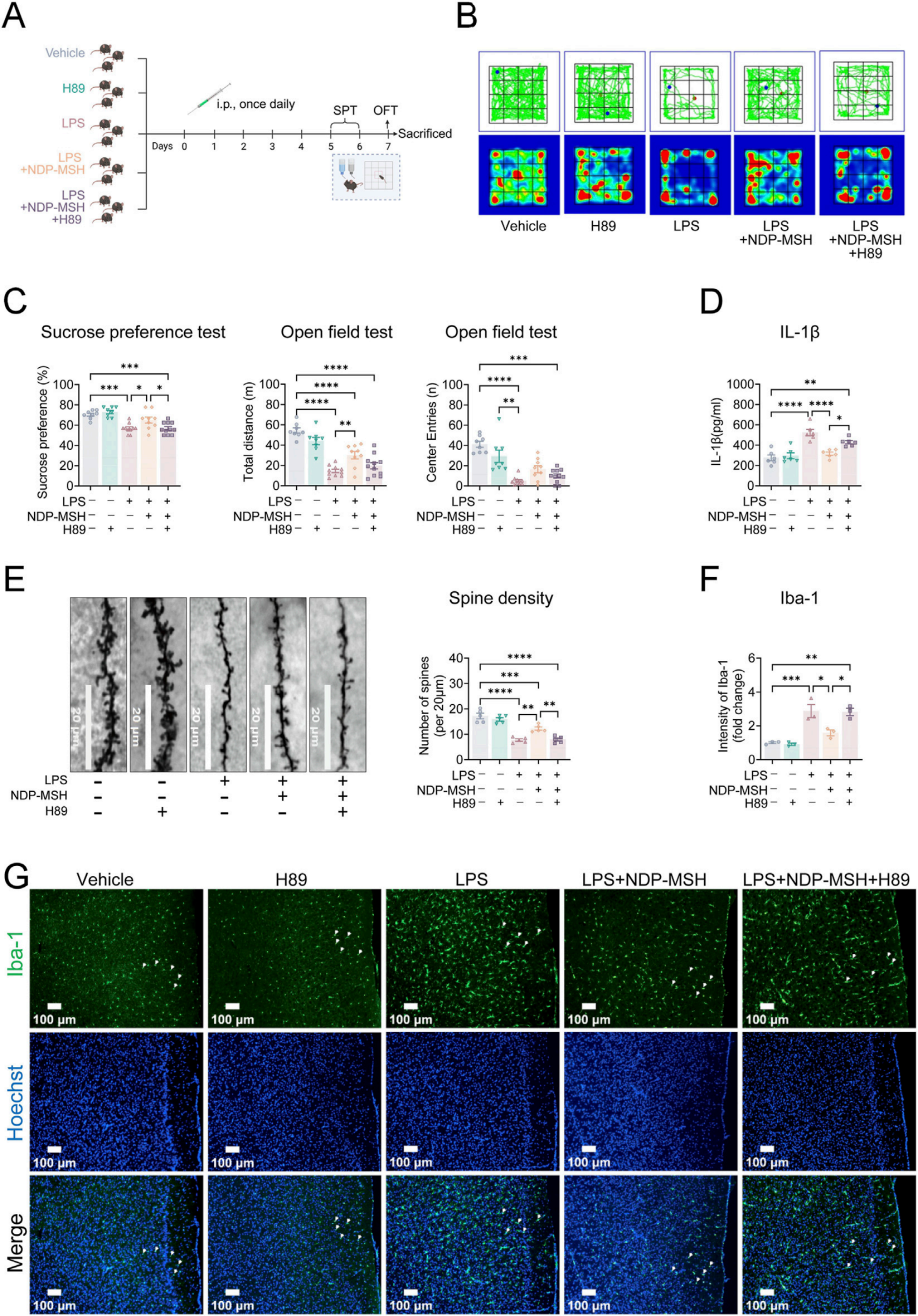
PKA inhibition by H89 abolishes the neuroprotective and antidepressant effects of NDP-MSH. **(A)** Experimental timeline and grouping strategy. Mice were assigned to Vehicle, LPS, LPS + NDP-MSH(NDP), LPS + NDP-MSH + H89(LNH), or H89 groups. **(B)** Representative locomotor traces in the OFT. **(C)** Behavioral assessments. NDP-MSH significantly increased sucrose preference in LPS-treated mice, whereas H89 co-treatment abolished this effect in the SPT. (One-way ANOVA, F_(4, 41)_ = 13.96, *p <* 0.0001, followed by Tukey’s multiple comparisons test. Vehicle vs. LPS: q_(41)_ = 6.649, *p <* 0.001; Vehicle vs. LNH: q_(41)_ = 6.804, *p <* 0.001; LPS vs. NDP: q_(41)_ = 4.128, *p <* 0.05; NDP vs. LNH: q_(41)_ = 4.237, *p <* 0.05). NDP-MSH restored total distance traveled reduced by LPS; H89 partly reversed this improvement in the OFT (One-way ANOVA, F_(4, 41)_ = 29.92, *p <* 0.0001, followed by Tukey’s multiple comparisons test. Vehicle vs. LPS: q_(41)_ = 13.07, *p <* 0.0001; Vehicle vs. NDP: q_(41)_ = 7.823, *p <* 0.0001; Vehicle vs. LNH: q_(41)_ = 11.660, *p <* 0.0001; LPS vs. NDP, q_(41)_ = 5.225, *p <* 0.01). NDP-MSH partly restored central zone entries reduced by LPS; H89 partly reversed this improvement in the OFT (Kruskal-Wallis non-parametric test, H_(4)_ = 30.23, *p* < 0.0001, followed by Dunn's multiple comparisons test. Vehicle vs. LPS: Z = 4.745, *p* < 0.0001; Vehicle vs. NDP: Z = 2.600, *p* = 0.093; Vehicle vs. LNH: Z = 3.924, *p* < 0.0001; H89 vs. LPS: Z = 3.615, *p* < 0.01) (Veh, n = 8; H89, n = 8; LPS, n = 10; NDP, n = 9; LNH, n = 11). **(D)** ELISA quantification of IL-1β levels in the PFC. NDP-MSH reversed LPS-induced IL-1β elevation, whereas H89 prevented this effect (One-way ANOVA, F_(4, 25)_ = 18.02, *p <* 0.0001, followed by Tukey’s multiple comparisons test. Vehicle vs. LPS, q_(25)_ = 9.990, *p <* 0.0001; Vehicle vs. LNH: q_(25)_ = 6.190, *p <* 0.01; LPS vs. NDP, q_(25)_ = 8.350, *p <* 0.0001; NDP vs. LNH, q_(25)_ = 4.549, *p <* 0.05) (n = 6 mice per group). **(E)** Representative images of mPFC neuronal dendrites by Golgi-Cox staining. Scale bar = 20 μm. Column graphs illustrate the relative spine numbers. NDP-MSH significantly increased spine density reduced by LPS treatment, whereas co-treatment with the PKA inhibitor H89 abolished this restorative effect (One-way ANOVA, F_(4, 20)_ = 36.61, *p <* 0.0001, followed by Tukey’s multiple comparisons test. Veh vs. LPS, q_(20)_ = 13.07, *p <* 0.0001; Veh vs. NDP, q_(20)_ = 6.820, *p <* 0.001; Veh vs. LNH, q_(20)_ = 12.82, *p <* 0.0001; LPS vs. NDP, q_(20)_ = 6.245, *p <* 0.01; NDP vs. LNH, q_(20)_ = 5.995, *p <* 0.01) (n = 5 mice per group, with 4-6 sections per mouse; at least 10 dendritic segments were randomly selected and quantified per section). **(F)** Quantification of relative Iba-1 fluorescence intensity, NDP-MSH normalized LPS-evoked microglial activation; H89 co-treatment restored microglial density to LPS-like levels (One-way ANOVA, F_(4, 10)_ = 19.57, *p <* 0.001, followed by Tukey’s multiple comparisons test. Veh vs. LPS, q_(10)_ = 8.650, *p <* 0.001; Veh vs. LNH, q_(10)_ = 8.412, *p <* 0.01; LPS vs. NDP, q_(10)_ = 5.940, *p <* 0.05; NDP vs. LNH, q_(10)_ = 5.702, *p <* 0.05) (n = 3 mice per group). **(G)** Representative immunofluorescence images of Iba-1 staining in the mPFC, Scale bar = 100 µm. Data are presented as mean ± SEM. *p < 0.05, **p < 0.01, ***p < 0.001.****p < 0.0001. Source: BioRender.com.

To determine whether the cAMP/PKA signaling pathway is required for the behavioral and neuroprotective actions of NDP-MSH, we pharmacologically inhibited PKA activity *in vivo* using H89 (Otmakhova et al., 2000), a selective PKA inhibitor. Mice were assigned to five groups following the experimental timeline shown in Figure 3A: Vehicle, H89, LPS, LPS + NDP-MSH, and LPS + NDP-MSH + H89. Representative exploratory tracks and heatmaps in the OFT illustrated that the locomotor recovery induced by NDP-MSH was noticeably suppressed by H89 co-administration (Figure 3B).

Behavioral assessments confirmed that NDP-MSH significantly increased sucrose preference in the SPT compared with the LPS group; importantly, co-administration of H89 completely abolished this antidepressant-like effect (Figure 3C, left). Similarly, in the OFT, NDP-MSH treatment markedly restored both the total distance traveled (Figure 3C, middle) and the number of center entries (Figure 3C, right), whereas H89 co-treatment robustly reversed these improvements back to LPS-like levels. Notably, H89 administration alone produced no detectable alterations in any behavioral parameters compared to the vehicle control. These data collectively demonstrate that pharmacological inhibition of PKA disrupts the antidepressant-like actions of NDP-MSH.

To evaluate whether PKA activity mediates the anti-inflammatory properties of NDP-MSH, IL-1β levels in the PFC were quantified via ELISA. LPS challenge markedly increased IL-1β expression, which was significantly attenuated by NDP-MSH treatment. However, co-administration of H89 blocked this anti-inflammatory effect, returning IL-1β concentrations to levels comparable to the LPS-only group (Figure 3D). Corroborating the inflammatory profile, immunofluorescence analysis of Iba-1 indicated that LPS exposure induced robust microglial activation in the PFC (Figures 3F,G). NDP-MSH treatment effectively normalized Iba-1 staining intensity, whereas H89 co-treatment completely prevented this suppression, resulting in sustained microglial activation similar to that of the LPS group (Figure 3F).

Finally, Golgi staining was performed to assess dendritic spine density in the PFC (Figure 3E). LPS exposure caused a severe reduction in spine density relative to the vehicle controls. NDP-MSH intervention significantly rescued this synaptic deficit, while H89 co-treatment entirely abolished the restorative effect, pulling spine density back down to LPS-like levels.

Taken together, these findings indicate that H89-mediated inhibition of PKA robustly abolishes the behavioral, anti-inflammatory, and synaptic protective benefits of NDP-MSH. This demonstrates that the cAMP/PKA signaling pathway is indispensable for the neuroprotective and antidepressant-like actions of MC1R activation.

### MC1R activation enhances synaptic transmission and intrinsic excitability in PFC neurons

**FIGURE 4:**
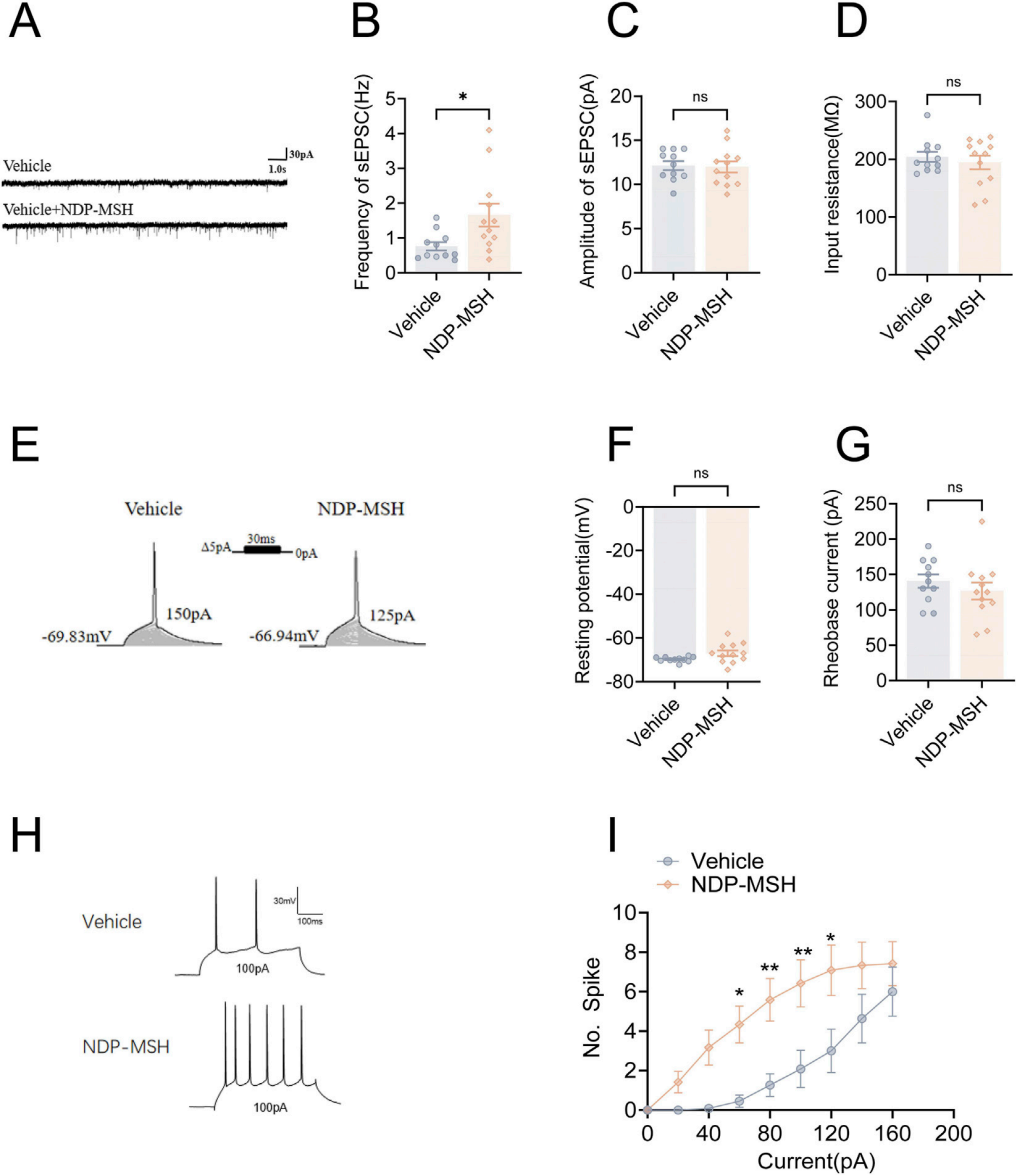
MC1R activation enhances synaptic transmission and intrinsic excitability in PFC neurons. **(A)** Representative traces of spontaneous excitatory postsynaptic currents (sEPSCs) recorded from mPFC pyramidal neurons. **(B)** Quantification of the frequency of sEPSCs in neurons from Vehicle- and NDP-MSH-treated mice. NDP-MSH significantly increased sEPSC frequency (two-tailed unpaired Student’s t-test, T_21_ = 2.473, *p* < 0.05) (Vehicle, n = 11 neurons; NDP-MSH, n = 12 neurons). **(C)** Quantification of sEPSC amplitude showing no significant difference between Vehicle and NDP-MSH groups (two-tailed unpaired Student’s t-test, T_21_ = 0.1817, *p* > 0.05) (Vehicle, n = 11 neurons; NDP-MSH, n = 12 neurons). **(D)** Comparison of input resistance (MΩ) between Vehicle- and NDP-MSH–treated neurons, with no significant change observed (two-tailed unpaired Student’s t-test, T_21_ = 0.6470, *p* > 0.05) (Vehicle, n = 11 neurons; NDP-MSH, n = 12 neurons). **(E)** Representative traces showing rheobase-evoked action potentials in neurons from Vehicle-treated (left) and NDP-MSH–treated (right) groups. The minimal current injection step (Δ5 pA, 30 ms) required to evoke the first spike is indicated, with corresponding rheobase currents and resting membrane potentials shown below traces. **(F)** Summary of resting membrane potential (RMP) in Vehicle and NDP-MSH groups. No significant difference was observed between groups (two-tailed unpaired Student’s t-test, T_21_ = 1.986, *p* > 0.05) (Vehicle, n = 11 neurons; NDP-MSH, n = 12 neurons). **(G)** Quantification of rheobase current showing comparable current thresholds for action potential initiation between Vehicle- and NDP-MSH–treated neurons (two-tailed unpaired Student’s t-test, T_21_ = 0.8929, *p* > 0.05) (Vehicle, n = 11 neurons; NDP-MSH, n = 12 neurons). **(H)** Representative current-clamp recordings showing action potentials evoked by a 100 pA current step in neurons from Vehicle-treated and NDP-MSH-treated groups. Neurons exposed to NDP-MSH displayed increased spike firing under the same current injection. **(I)** Frequency-current **(F–I)** relationship showing evoked spike numbers in response to increasing depolarizing current injections in Vehicle and NDP-MSH groups. Two-way repeated-measures ANOVA revealed a significant Current × Treatment interaction (F_(8, 168)_ = 3.600, *p* < 0.001) and a significant main effect of treatment (F_(1, 21)_ = 7.456, *p* < 0.05), indicating that NDP-MSH increased spike output compared with Vehicle. Šídák’s multiple comparisons test showed significant differences between Vehicle and NDP-MSH groups at the indicated current intensities (*p < 0.05, **p < 0.01) (Vehicle, n = 11 neurons; NDP-MSH, n = 12 neurons). Data are presented as mean ± SEM. Statistical significance: *p < 0.05, **p < 0.01. Source: BioRender.com.

Given our structural findings that NDP-MSH preserves dendritic spine density and reverses synaptic protein deficits, we next sought to investigate whether MC1R activation directly modulates functional synaptic transmission and neuronal excitability in the PFC using whole-cell patch-clamp recordings (Figure 4).

We first evaluated spontaneous excitatory postsynaptic currents (sEPSCs) to assess functional synaptic strength (Figure 4A). Pharmacological activation of MC1R by NDP-MSH led to a significant increase in the frequency of sEPSCs compared with the vehicle control group (Figure 4B). In contrast, the mean amplitude of sEPSCs remained unaltered between the two groups (Figure 4C), suggesting that MC1R activation predominantly enhances presynaptic glutamate release probability rather than altering postsynaptic receptor responsiveness under baseline conditions. Concurrently, no significant differences were observed in basic passive membrane properties, including input resistance (Figure 4D) and resting membrane potential (Figure 4F), between vehicle- and NDP-MSH-treated neurons.

To further characterize how MC1R regulatory mechanisms influence intrinsic neuronal excitability, we examined action potential firing properties. While the rheobase current (the minimum current required to trigger an action potential) showed a downward trend in the NDP-MSH group, the difference did not reach statistical significance (Figures 4E,G). However, when subjected to a series of depolarizing current injections (Figure 4H), neurons treated with NDP-MSH exhibited a significantly higher number of spikes (action potentials) across multiple current steps compared to vehicle-treated neurons, as illustrated by the shifted input-output curve (Figure 4I).

Taken together, these electrophysiological data demonstrate that NDP-MSH administration significantly boosts both excitatory synaptic transmission and the intrinsic firing excitability of PFC neurons. This functional enhancement provides a robust physiological mechanism that potentially underlies the protective effects of NDP-MSH against LPS-induced synaptic loss and signaling impairments.

### NDP-MSH normalizes LPS-induced metabolic reprogramming through cAMP/PKA signaling

To further elucidate the mechanisms by which NDP-MSH alleviates LPS-induced depressive-like behaviors, untargeted metabolomic profiling of the prefrontal cortex was performed across different treatment groups. The experimental groups included: control (Ctrl, vehicle-treated mice), LPS (mice injected with LPS), NDP (mice co-treated with LPS and NDP-MSH), LNH (mice co-treated with LPS, NDP-MSH, and the PKA inhibitor H89), and H89 (mice treated with H89 alone).

**FIGURE 5:**
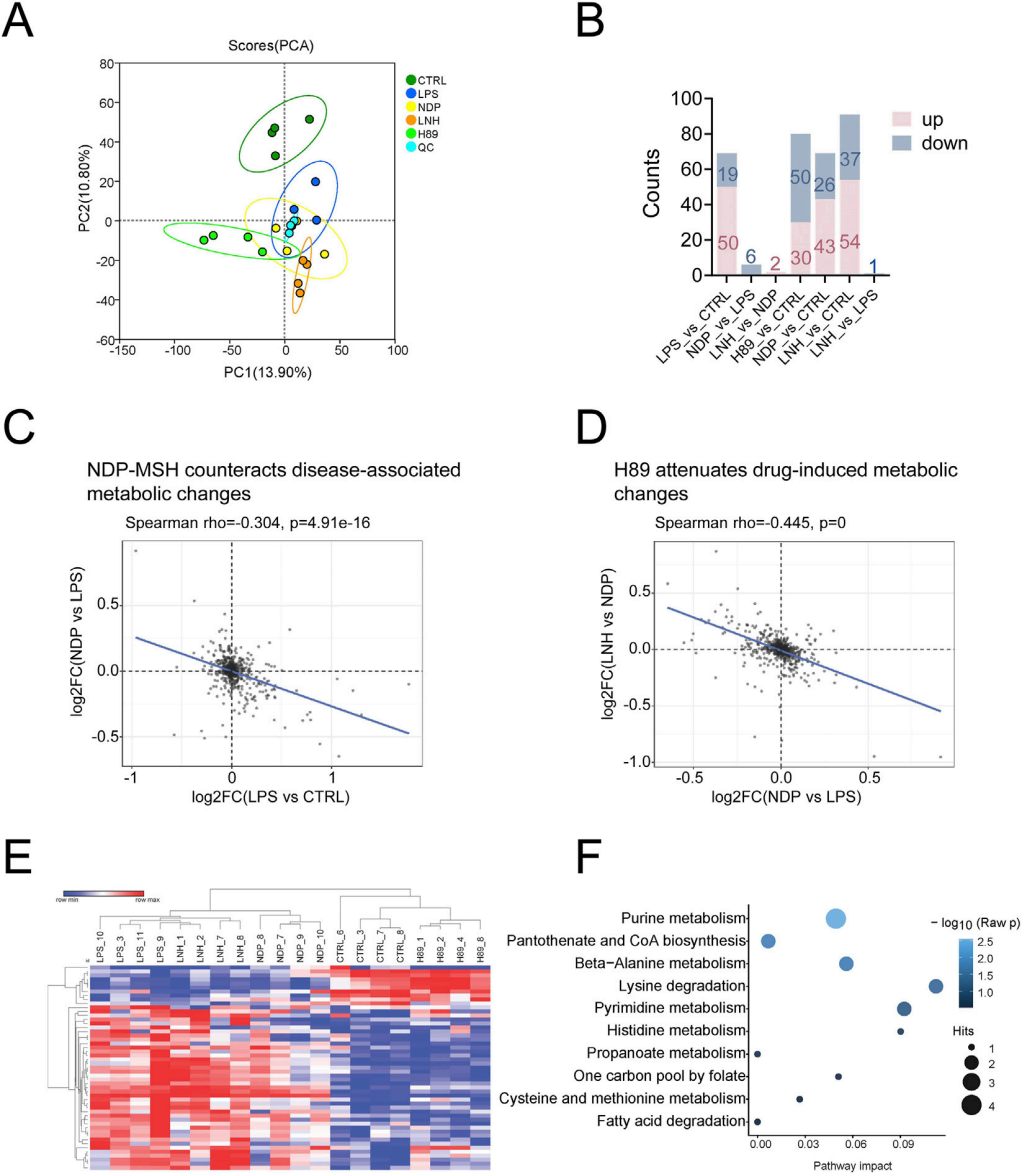
NDP-MSH Normalizes LPS-induced Metabolic Reprogramming through cAMP/PKA Signaling. **(A)** Principal component analysis (PCA) score plot of untargeted metabolomic profiles from saline control (CTRL), LPS-treated (LPS), drug-treated (NDP), drug plus inhibitor-treated (LNH), inhibitor alone (H89), and quality control (QC) samples. Biological samples included n = 4 mice per treatment group (CTRL, LPS, NDP, LNH, and H89). QC samples (n = 3) were used for quality control and were excluded from statistical comparisons. **(B)** Bar plot showing the number of significantly altered metabolites (upregulated and downregulated) across pairwise comparisons (limma, FDR <0.2, |log_2_ fold change| ≥ 0.15; QC samples excluded). **(C)** Scatter plot showing the correlation between LPS-induced metabolic changes (LPS vs. CTRL) and drug-induced changes (NDP vs. LPS). **(D)** Scatter plot showing the correlation between drug-induced changes (NDP vs. LPS) and inhibitor-on-drug effects (LNH vs. NDP). **(E)** Heatmap showing a selected subset of metabolites whose LPS-induced changes were reversed by NDP-MSH treatment and whose NDP-MSH-mediated reversal was blocked by H89. Relative metabolite intensities were Z-score normalized across group means. **(F)** KEGG pathway enrichment analysis of drug-responsive metabolites. Source: BioRender.com.

A total of 688 metabolites were detected, including 460 in positive-ion mode and 228 in negative-ion mode (Supplementary Table S1). Principal component analysis (PCA) revealed a clear separation between control and LPS-treated samples, indicating that LPS exposure induced a robust global metabolic reprogramming (Figure 5A). Consistently, differential metabolite analysis confirmed that LPS treatment resulted in widespread alterations in metabolite abundance compared with controls. Quality control (QC) samples clustered tightly in PCA space, indicating stable analytical performance during data acquisition. In addition, the RSD distribution of detected features across QC injections showed that 94.52% of raw QC features and 98.27% of preprocessed QC features had an RSD <30% (Supplementary Figure S1), further supporting the reproducibility of the metabolomics platform. We further quantified the number of differentially altered metabolites between each pairwise group comparison, as summarized in Figure 5B.

To assess whether NDP-MSH counteracted LPS-induced metabolic disturbances, we compared log_2_ fold changes of metabolites identified in the LPS vs. Ctrl comparison with those in the NDP vs. LPS comparison. This analysis revealed a significant negative correlation (Spearman ρ ≈ − 0.30, *p* < 1 × 10^−15^), indicating that metabolites dysregulated by LPS tended to shift in the opposite direction following NDP-MSH treatment (Figure 5C). To determine whether these metabolic effects were mediated via the cAMP/PKA signaling pathway, H89 was co-administered with NDP-MSH (LNH group). Notably, comparison of log_2_ fold changes between NDP vs. LPS and LNH vs. NDP revealed an even stronger negative correlation (Spearman ρ ≈ −0.45, *p* < 1 × 10^−16^) (Figure 5D), suggesting that H89 broadly attenuated NDP-MSH-induced metabolic corrections. Together, these results indicate that NDP-MSH globally reverses disease-associated metabolic alterations induced by LPS, and that this metabolic reprogramming is largely mediated through the cAMP/PKA pathway.

To identify specific downstream metabolic pathways associated with these global shifts, we screened for a subset of metabolites that met a stringent set of criteria: significantly altered by LPS, showing a directionally consistent reversal after NDP-MSH treatment, and exhibiting an opposite shift after H89 administration, suggestive of attenuation of the NDP-MSH-mediated metabolic response. A hierarchical clustering heatmap of these responsive metabolites displayed tightly coordinated expression patterns across the five experimental groups (Figure 5E). Pathway enrichment analysis on this subset revealed that these metabolites were predominantly clustered into purine metabolism, pantothenate and CoA biosynthesis, and multiple amino acid pathways (such as lysine degradation and beta-alanine metabolism) (Figure 5F). Intriguingly, these enriched pathways provide a direct metabolic scaffolding that aligns with our observed synaptic and inflammatory phenotypes. Specifically, the remodeling of purine metabolism is highly continuous with the energetic demands of enhanced intrinsic neuronal firing and the regulation of microglial neuroinflammation (Fields and Burnstock, 2006; Illes et al., 2020). Furthermore, the normalization of pantothenate and CoA biosynthesis yields critical substrates required for mitochondrial bioenergetics (Leonardi and Jackowski, 2007) and lipid synthesis (Czumaj et al., 2020), both of which are metabolic prerequisites for preserving dendritic spine density and synaptic protein scaffolding (such as PSD95 and GluA1) (Kreienkamp, 2008).

Collectively, these findings suggest a coordinated, systems-level metabolic response to inflammatory challenge and pharmacological intervention, highlighting metabolic network remodeling as a key component of NDP-MSH–mediated antidepressant effects.

### mPFC-specific overexpression of *Mc1r* rescues LPS-induced depressive-like behaviors

**FIGURE 6:**
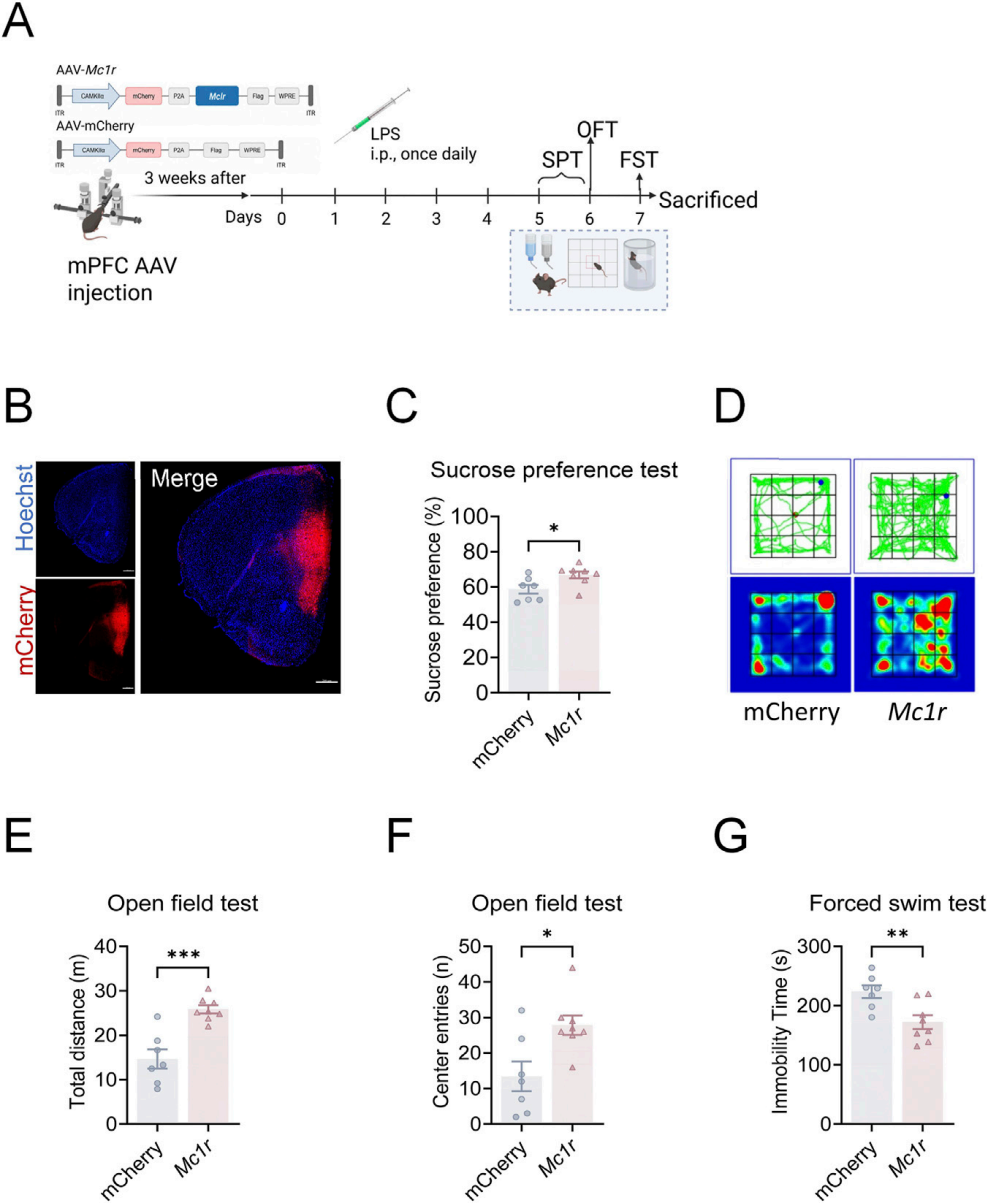
Overexpression of *Mc1r* in the medial prefrontal cortex alleviates LPS-induced depressive-like behaviors. **(A)** The schematic representation of the viral constructs and the experimental timeline. Bilateral AAV injection into the medial prefrontal cortex (mPFC), followed by a 3-week expression period prior to lipopolysaccharide (LPS; 1 mg/kg, i. p., once daily) administration. Behavioral tests, including the SPT, OFT, and FST, were conducted on days 5–7, after which mice were sacrificed for tissue collection. **(B)** Representative fluorescence images showing mCherry expression in the mPFC following AAV injection. Merged images confirm robust and localized expression within the mPFC. Scale bar = 200 μm. **(C)** Sucrose preference in the SPT. LPS-treated mice exhibited a significantly reduced sucrose preference compared with *Mc1r* overexpression group (two-tailed unpaired Student’s t-test; T_13_ = 2.595, *p <* 0.05) (LPS + mCherry, n = 7; LPS + AAV-*Mc1r*, n = 8). **(D)** Representative locomotor trajectories (upper panels) and corresponding heat maps (lower panels) from the OFT in LPS-treated mice expressing control virus (LPS + mCherry) and LPS-treated mice with *Mc1r* overexpression (LPS + AAV-*Mc1r*). **(E)** Total distance traveled in the OFT. *Mc1r* overexpression attenuated the LPS-induced reduction in total distance traveled (two-tailed unpaired Student’s t-test; T_13_ = 5.019, *p <* 0.001) (LPS + mCherry, n = 7; LPS + AAV-*Mc1r*, n = 8). **(F)** Number of center entries in the OFT. *Mc1r* overexpression attenuated the LPS-induced reduction center entries (two-tailed unpaired Student’s t-test; T_13_ = 2.952, *p <* 0.05) (LPS + mCherry, n = 7; LPS + AAV- *Mc1r*, n = 8). **(G)** Immobility time in the FST. Overexpression of *Mc1r* significantly reduced immobility time compared with the LPS-treated mice (two-tailed unpaired Student’s t-test; T_13_ = 3.192, *p <* 0.01) (LPS + mCherry, n = 7; LPS + AAV- *Mc1r*, n = 8). Data are presented as mean ± SEM. Statistical significance: *p < 0.05, **p < 0.01, ***p < 0.001. Source: BioRender.com.

Since NDP-MSH is a pan-melanocortin receptor agonist capable of activating multiple receptor subtypes (including MC1R, MC3R, MC4R, and MC5R), we next investigated whether the observed antidepressant effects are mediated specifically through MC1R. Given that the medial prefrontal cortex (mPFC) plays a central role in the regulation of emotion and the pathogenesis of depressive-like behaviors (Covington et al., 2010; Wang et al., 2022), we focused our investigation on this specific subregion and employed an adeno-associated virus (AAV) strategy to selectively overexpress *Mc1r* within the mPFC. Recombinant AAVs encoding *Mc1r* (AAV-*Mc1r*) or a control fluorophore (AAV-mCherry) driven by the CaMKIIα promoter were stereotaxically microinjected into the mPFC. The schematic representation of the viral constructs and the experimental timeline are illustrated in Figure 6A.

Three weeks following AAV microinjection to allow for robust viral expression, mice were subjected to repeated LPS administration followed by behavioral assessments. Successful viral targeting and localized expression within the mPFC were verified by the detection of mCherry fluorescence (Figure 6B). Behavioral analyses revealed that mice in the *Mc1r*-overexpression group (LPS + AAV-*Mc1r*) exhibited a significant restoration of sucrose preference in the SPT compared with the control group (LPS + AAV-mCherry) (Figure 6C). In the OFT, representative exploratory tracks and heatmaps indicated a marked improvement in locomotor and exploratory activities in the AAV-*Mc1r* group (Figure 6D). Quantitative analysis confirmed that *Mc1r* overexpression robustly rescued the LPS-induced behavioral suppression, markedly restoring the total distance traveled (Figure 6E) and the number of center entries (Figure 6F) in the OFT. Furthermore, in the forced swim test (FST), mice with *Mc1r* overexpression displayed a reduction in immobility time relative to the control mice (Figure 6G).

Taken together, these results demonstrate that mPFC-specific overexpression of *Mc1r* reverses LPS-induced inflammation-associated depressive-like behaviors, establishing a critical and causal role for localized MC1R signaling within this cortical subregion. Corroborating our pharmacological evidence, these genetic findings firmly indicate that the activation of the mPFC MC1R-cAMP/PKA signaling pathway is a key mechanism mediating the mitigation of neuroinflammation and depressive-like phenotypes.

## Discussion

In this study, MC1R expression was decreased in the prefrontal cortex (PFC) of mice exhibiting LPS-induced inflammation-associated depressive-like behaviors. Administration of the MC1R ligand analog NDP-MSH reversed these depressive-like behaviors and suppressed neuroinflammatory responses. These effects were accompanied by increased PKA phosphorylation. Mechanistically, pharmacological inhibition of cAMP/PKA signaling with H89 abolished the behavioral, neuroimmune, and metabolomic effects of NDP-MSH. Genetically, overexpression of MC1R in the mPFC was sufficient to reverse LPS-induced behavioral deficits. Collectively, these findings indicate that NDP-MSH alleviates inflammation-associated depressive-like behaviors in association with melanocortin signaling involving MC1R and downstream cAMP/PKA activation in the PFC.

The cAMP/PKA signaling is a central pathway implicated in the pathophysiology of depression and in the mechanisms of antidepressant action, influencing affective behaviors through regulation of neuroplasticity and inflammatory processes (Yang et al., 2025). Clinical evidence indicates that cAMP levels are reduced in the brains of patients with depression(Perez et al., 2002), and that expression of multiple PKA subunits is decreased in postmortem brain tissue from individuals with depressive disorders (Shelton et al., 2009). In addition, antidepressant treatment has been shown to restore impaired cAMP signaling in depressed patients (Gao et al., 2022). Preclinical studies further demonstrate that chronic stress–based models lead to suppression of cAMP/PKA/CREB signaling accompanied by deficits in neuroplasticity and affective behaviors (Wang et al., 2015). Notably, many physiological and pathological functions of MC1R in both melanocytic and non-melanocytic cells are mediated by cAMP signaling (Li and Taylor, 2008; D'Orazio and Fisher, 2011). In line with this literature, our findings indicate that NDP-MSH is associated with reduced neuroinflammatory responses and improvements in depressive-like behaviors, in a cAMP/PKA-dependent manner. However, the downstream cellular processes through which cAMP/PKA signaling contributes to behavioral improvement remain incompletely understood.

Accumulating evidence suggests that cAMP/PKA signaling plays a critical role in the regulation of excitatory synaptic transmission. Early electrophysiological studies demonstrated that manipulation of cAMP/PKA signaling alters excitatory postsynaptic potentials and modulates excitatory synaptic transmission (Colwell and Levine, 1995). Subsequent work revealed that neuronal cAMP/PKA signaling regulates synaptic vesicle release (Park et al., 2014) and controls the phosphorylation state of NMDA receptors (Skeberdis et al., 2006) and AMPA-type ionotropic glutamate receptors (Zhao et al., 2019), thereby enhancing synaptic efficacy and plasticity (Abel and Nguyen, 2008). In the present study, patch-clamp recordings demonstrated that NDP-MSH enhanced excitatory synaptic transmission. Consistent with these electrophysiological findings, Western blot analysis showed that NDP-MSH significantly increased the expression of PSD95 and the AMPA receptor subunit GluA1 in the prefrontal cortex. In addition, Golgi-Cox staining showed that NDP-MSH restored the LPS-induced reduction in dendritic spine density, providing structural evidence supporting its synaptic protective effect. These findings suggest that melanocortin signaling involving MC1R and downstream cAMP/PKA activation may support the recovery of excitatory synaptic function under inflammatory conditions. Given that reduced cortical excitatory synaptic activity is a recognized feature of depression, enhancement of excitatory synaptic transmission under inflammatory conditions may represent one mechanism through which NDP-MSH attenuates depressive-like behaviors.

Our metabolomic analyses revealed enrichment of pathways related to metabolic processes such as purine metabolism, pantothenate and coenzyme A (CoA) biosynthesis, and beta-alanine metabolism. Among these pathways, purine metabolism emerged as a major node, consistent with the requirement of ATP as the substrate for cAMP synthesis (Huang et al., 2021). Disruption of purine metabolism under inflammatory conditions (Linden et al., 2019; Qian et al., 2024) may therefore constrain cAMP/PKA signaling capacity, whereas normalization of this pathway following NDP-MSH treatment is consistent with sustained activation of PKA signaling and the energetic support for neuronal intrinsic firing. In addition, purinergic metabolites are well-established modulators of neuroimmune signaling (Calovi et al., 2019; Illes et al., 2020), suggesting that restoration of nucleotide homeostasis may contribute to the observed attenuation of neuroinflammation in the prefrontal cortex.

Pathways related to pantothenate and CoA biosynthesis further suggests alterations in CoA availability, a key determinant of mitochondrial function and cellular energy balance (Leonardi et al., 2005). As adequate energy supply is required for both inflammatory regulation and excitatory synaptic transmission (Harris et al., 2012; O'Neill et al., 2016), normalization of CoA-related metabolism may establish a metabolic context permissive for cAMP/PKA-dependent signaling and functional recovery of neural synaptic drive and intrinsic excitability. This metabolic stabilization aligns with the enhanced expression of the synaptic proteins PSD95 and GluA1 observed in our study. Supporting this interpretation, other metabolic pathways involved in energy and nucleotide homeostasis, including beta-alanine (Leonardi et al., 2005), propanoate (Martínez-Reyes and Chandel, 2020), and pyrimidine (Lane and Fan, 2015) metabolism, were also identified through pathway enrichment analysis. Collectively, these findings indicate that MC1R–cAMP/PKA signaling is associated with broader immunometabolic adaptations that may be associated with suppression of neuroinflammation and restoration of excitatory synaptic transmission, rather than acting through a single synapse-specific metabolic pathway. Further investigation is warranted to delineate the precise mechanisms underlying these adaptations.

Several limitations of the present study should be acknowledged. First, the repeated systemic LPS model primarily reflects inflammation-associated behavioral alterations and does not fully recapitulate the psychological and biological complexity of human depressive disorders. Because systemic LPS can induce sickness-related responses, reduced locomotor activity may coexist with depressive-like behavioral changes and should be considered when interpreting OFT-based measures. Thus, our behavioral interpretation was based on the combined profile of sucrose preference, locomotor/exploratory activity, and neurobiological alterations in the PFC, rather than on OFT performance alone. Future studies using chronic stress-based models will be useful to further evaluate the long-term efficacy of NDP-MSH-related interventions in depression-relevant conditions. Second, because NDP-MSH is not an MC1R-selective agonist, possible contributions from other melanocortin receptor subtypes cannot be fully excluded. Although mPFC-specific Mc1r overexpression supports the functional involvement of MC1R signaling in this model, future studies using MC1R-selective antagonists or MC1R knockout mice will be required to determine the receptor subtype specificity of NDP-MSH more precisely. Finally, the untargeted metabolomics analysis was designed as an exploratory pathway-level assessment. Therefore, the identified metabolic alterations should be interpreted as pathway-level changes rather than validated metabolic biomarkers. Future targeted quantitative LC-MS/MS analyses will be needed to validate specific metabolites within the purine metabolism, pantothenate and CoA biosynthesis, and related pathways.

In summary, the present study demonstrates that NDP-MSH exerts anti-inflammatory and neuroprotective effects in an inflammation-associated depression model through activation of MC1R and cAMP/PKA signaling. These findings highlighted that MC1R as a key immunomodulatory node that integrates neuroinflammation, synaptic function, and affective behavior, and further suggest that NDP-MSH may represent a promising candidate for further development as a therapeutic strategy for neuroinflammation-related depression.

## Data Availability

The untargeted metabolomics datasets generated and analyzed during the current study are publicly available in the Mendeley Data repository at https://data.mendeley.com/ with the DOI:10.17632/8rsxrhfp68.1.
